# The effect of antihypertensive treatment on longitudinal changes in PLGF and sFlt‐1 in women with new onset hypertension in pregnancy

**DOI:** 10.1111/aogs.70221

**Published:** 2026-05-13

**Authors:** Edward Tyrell, Katherine G. Y. Lau, Martyna Bednorz, Kypros H. Nicolaides, Nikos A. Kametas

**Affiliations:** ^1^ Antenatal Hypertension Clinic King's College Hospital London UK; ^2^ King's College London University of London London UK; ^3^ Fetal Medicine Research Institute King's College Hospital London UK

**Keywords:** angiogenic factors, antihypertensive treatment, hypertension, longitudinal changes, placental growth factor, pre‐eclampsia, pregnancy, soluble fms‐like tyrosine kinase‐1

## Abstract

**Introduction:**

To assess longitudinal changes in serum sFlt‐1 and PLGF in women with newly diagnosed hypertension in pregnancy before and after commencing antihypertensive therapy, aiming at tight BP control (<135/85 mmHg).

**Material and Methods:**

This was a prospective study that assessed women at presentation with hypertension and after 1 and 2 weeks of antihypertensive therapy. At each visit, blood pressure (BP) and blood tests to obtain levels of sFlt‐1 and PLGF were taken. A multilevel linear mixed‐effects model was performed to compare the repeated measurements of sFlt‐1 and PLGF, controlling for maternal demographics, diagnosis at presentation, and antihypertensive agent used. The cohort was further subdivided into two groups based on how quickly they achieved target BP control to assess whether the degree of initial control influenced the angiogenic profile. Group 1 achieved BP < 135/85 after 1 week, while group 2 after 2 weeks of treatment.

**Results:**

A total of 133 women were recruited into the study, 47 in group 1 and 86 in group 2. For the total cohort, both systolic blood pressure (SBP) and diastolic blood pressure (DBP) dropped after 1 week on antihypertensive medication (*p* < 0.001) and remained stable thereafter. Log_10_PLGF demonstrated a continuous fall (p < 0.001) while Log_10_sFlt‐1 did not change after 1 and 2 weeks on medication.

Group 1 had persistently higher Log_10_PLGF than group 2 (*p* = 0.015). Both groups showed a reduction in Log_10_PLGF at a similar rate over 2 weeks on antihypertensive medication. There was no difference between groups throughout the study in Log_10_sFlt‐1 and no change over time. There was a significant interaction between groups for SBP and DBP (*p* < 0.001) but no interaction between groups for Log_10_PLGF or Log_10_ sFlt‐1.

**Conclusions:**

In pregnant women with new‐onset hypertension, tight BP control leads to a reduction in SBP and DBP within the first week of antihypertensive treatment. Log_10_PLGF declines, but Log_10_sFlt‐1 shows no change with time. Women who achieved BP control within 1 week, compared to 2 weeks from commencing treatment, had higher Log_10_PLGF but no difference in Log_10_ sFlt‐1. Both groups demonstrated similar changes, with Log_10_PLGF declining but Log_10_sFlt‐1 remaining static with time.

## Graphical abstract

In pregnant women with new‐onset hypertension starting antihypertensives, some achieve tight BP control within a week (Group 1‐blue line), while others respond slower (Group 2‐red line). Group 1 showed higher Log10PLGF levels, but Log10sFlt‐1 did not differ between groups. Over time, Log10PLGF declined in both groups, while Log10sFlt‐1 remained stable.

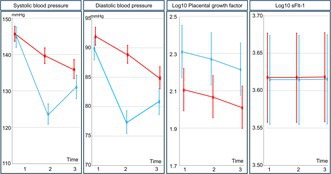

**Abbreviations**
BP: blood pressure
DBP: diastolic blood pressure
GH: gestational hypertension
PE: pre‐eclampsia
PLGF: placental‐like growth factor
SBP: systolic blood pressure
sFlt‐1: soluble fms‐like tyrosine kinase‐1

**Key message**

Serum PLGF and sFlt‐1 levels in pregnant women with new onset hypertension are affected by antihypertensive therapy.

## 1 INTRODUCTION

Hypertensive disorders in pregnancy affect 10% of pregnancies worldwide.^1^ They result in significant maternal and perinatal mortality and morbidity.^1^ Imbalance in pro‐angiogenic and anti‐angiogenic factors, including placental growth factor (PLGF) and soluble fms‐like tyrosine kinase‐1 (sFlt‐1), has been implicated in the pathophysiology of pre‐eclampsia (PE).^2^, ^3^, ^4^, ^5^ Tissue hypoxia in PE causes the placenta to release anti‐angiogenic factors, such as sFlt‐1, into the maternal circulation^6^ with a concomitant decrease in levels of angiogenic factors, such as vascular endothelial growth factor and PLGF.^7^, ^8^, ^9^ Lower PLGF and increased sFlt‐1 are associated with increased disease severity,^10^, ^11^ while an improved angiogenic profile may reduce end organ damage. This has been seen in mouse models with induced PE, where a reduction of sFlt‐1 has been shown to reduce glomerular damage.^12^

Two randomized control trials, the Control of Hypertension in Pregnancy and the Treatment of Mild Chronic Hypertension during Pregnancy trials, have demonstrated the benefits of tight blood pressure (BP) control in pregnancy including reduced episodes of severe maternal hypertension, preterm delivery, and a reduction in composite severe maternal outcomes.^13^, ^14^ The notion that tight BP control is associated with improved maternal outcomes raises the question whether antenatal BP control leads also to an improvement in angiogenic profile. In nonpregnant populations treatment with antihypertensives has been shown to reduce levels of both sFlt‐1 and VEGF.^15^ In pregnancy, small studies have shown a reduction in sFlt‐1 in women with PE treated with alpha methyldopa^16^, ^17^ and in women at high risk of PE treatment with atenolol led to a blunted rise in sFlt‐1.^18^ In vitro studies have posited varying effects on sFlt‐1 and PLGF production and their gene expression depending on which antihypertensive medication is used.^19^, ^20^, ^21^, ^22^ However, there are no studies examining the longitudinal changes in PLGF and sFlt‐1 in pregnant women with new‐onset hypertension, naïve to antihypertensive treatment who are managed with an aim to achieve tight BP control. We hypothesize that firstly: antihypertensive treatment and tight BP control may influence changes in sFlt‐1 and PLGF over time, and secondly, whether the response to treatment is related to initial serum values.

This study aimed to assess, in women with newly diagnosed hypertension in pregnancy, longitudinal changes of PLGF and sFlt‐1 at presentation and 1 and 2 weeks after initiation of antihypertensive therapy. In addition, we aimed to examine if the degree of BP control in the first week after initiation of antihypertensive therapy was mirrored in a difference in levels of PLGF and sFlt‐1. Hence, we divided our population into two groups depending on whether they reached target BP after 1 week of antihypertensive therapy to assess if swiftly achieving tight BP control affects PLGF and sFlt‐1 levels.

## 2 MATERIAL AND METHODS

### 2.1 Study population

This was a prospective study conducted in the Antenatal Hypertension Clinic at King's College Hospital, London between April 2018 and November 2023. This is a clinic dedicated to managing pregnancies in women with hypertensive disorders of pregnancy. For the current study, we included women with singleton pregnancies presenting with newly diagnosed hypertension after 20 weeks of gestation (gestational hypertension or pre‐eclampsia), who had at least 3 visits from presentation to delivery. Women had to be treatment naïve at the first visit and had to have had an two additional visits after commencement of antihypertensive treatment. Women with chronic (pre‐existing) hypertension were excluded. Gestational age was assessed by the measurement of fetal crown–rump length at 11–13 weeks.^23^, ^24^

The study participants were followed up weekly after the first presentation. Their management was according to local protocols. This included, at the first visit, the recording of maternal demographic characteristics and medical history. From the first visit and then at weekly intervals, BP was measured using a standardized protocol^25^ and an automated pregnancy validated device^26^ and maternal blood samples for full blood count, urea and electrolytes, liver function tests, and PLGF and sFlt‐1 were collected. In addition, ultrasound examination for fetal growth and Doppler assessment of impedance to flow in the uterine arteries, umbilical arteries, and middle cerebral arteries was performed. Serum levels of PLGF and sFlt‐1 were measured using an automated biochemical analyzer (BRAHMS KRYPTOR compact PLUS, Thermo Fisher Scientific, Hennigsdorf, Germany). The operators and clinicians managing the cases were blinded to the values of sFlt‐1 and PLGF.

### 2.2 Definitions and management

Diagnosis at presentation was made according to the International Society for the Study of Hypertension in Pregnancy (ISSHP) definitions.^27^ Gestational Hypertension (GH) was defined as hypertension with systolic blood pressure (SBP) ≥140 mmHg and/or diastolic blood pressure (DPB) ≥90 mmHg on at least two occasions more than 4 h apart, at a gestation >20 weeks. PE is defined as hypertension in the presence of proteinuria (24 h urine collection ≥300 mg or protein creatinine ratio ≥ 30 mg), evidence of end organ dysfunction (neurological dysfunction, pulmonary edema, hematological dysfunction such as platelets <150 000/ μ/L, disseminated intravascular coagulation or hemolysis, acute kidney injury with creatinine >90 μmol/L, liver involvement with alanine aminotransferase or aspartate aminotransferase >40 IU/L), or uteroplacental dysfunction.

Women were treated according to the level of their hypertension and clinical symptoms. First‐line antihypertensive agents included beta‐blockers (Labetalol or Atenolol) and vasodilatory agents (slow‐release Nifedipine or Methyldopa). The aim of antihypertensive treatment was a BP <135/85, reflecting tight control in the CHIPS randomized trial.^13^ After 1 week of treatment, women who had achieved the target BP were labeled as Group 1 and those who were slower to achieve the same target were labeled as Group 2.

### 2.3 Statistical analysis

The Kolmogoroff–Smirnoff test was used to assess normal distribution of the data. Data were expressed as median (interquartile range (IQR)) for continuous variables and n (%) for categorical variables. Differences between Groups 1 and 2 were assessed by the Mann–Whitney U test for numerical variables and chi‐squared test for categorical variables, respectively.

The distribution of PLGF and sFlt‐1 was made Gaussian after log10 transformation. A multilevel linear mixed‐effects model was used for the repeated measures analysis for systolic BP, diastolic BP, Log_10_PLGF and Log_10_sFlt‐1 over the three visits. We controlled for demographic and pregnancy characteristics as these are known to influence these angiogenic markers.^28^ These included maternal age, height and weight, smoking (yes/no), gestational age, parity (nulliparous, multiparous), race (white, black, other), type of antihypertensive agent (beta blocker, vasodilator), diagnosis at presentation (GH, PE), and aspirin. Low molecular weight heparin was omitted as this had not been administered in any patients in our cohort. The fixed‐effect component included time (the three visits), study group (groups 1 and 2), maternal age, height and weight, smoking, gestational age, parity, race, type of antihypertensive agent, aspirin, diagnosis at presentation and first‐order interaction between time and group. The likelihood ratio (LR) test was used to define the best multilevel model (including only the random slope for time or random intercept versus including both the random intercept and slope) and to compare it with the base model (with no random effects). The estimated marginal means of systolic BP, diastolic BP, Log_10_PLGF and Log_10_sFlt‐1 at each group/time combination are presented. The results of multilevel mixed effects models are presented in Tables S1 and S2 with only significant variables remaining in the final model.

Statistical analysis was carried out using SPSS (IBM SPSS Statistics for Windows, Version 29.0.2.0, Armonk, NY: IBM Corp).

## 3 RESULTS

### 3.1 Study population

The study population characteristics are shown in Table 1. A total of 133 women were recruited into the study, 47 in group 1 and 86 in group 2. There were no statistically significant differences between the two BP control groups in terms of age, height, weight, gestational age, parity, smoking status, initial systolic and diastolic BP, diagnosis at presentation, type of antihypertensive agent initiated, and serum sFlt‐1 levels. There were no women on low molecular weight heparin. Overall, 30% of women were on aspirin during pregnancy, 53% in group 1 and 18.6% in group 2. Women in groups 1 and 2 had similar BP at presentation. The same methodology for initiation and tailoring antihypertensive medication was used in both groups. Significant differences between the two groups were seen for PLGF (134.7 vs. 102.8, *p* = 0.015).

**TABLE 1:** Study population characteristics: maternal demographic characteristics, diagnosis, blood pressure (BP), medication commenced, and angiogenic factors at presentation.

	Total cohort (*n* = 133)	Group 1 (*n* = 47)	Group 2 (*n* = 86)	*p*‐value
Age (years)	35.0 (32.0–38.0)	35.0 (31.5–38.0)	35.0 (31.0–38.5)	0.420
Height (m)	166.0 (162.0–170.7)	166.0 (162.9–169.2)	166.0 (161.2–171.7)	0.994
Weight (kg)	76.0 (63.3–90.7)	76.0 (63.8–90.7)	79.1 (64.1–91.5)	0.656
Gestational Age (weeks)	35.3 (32.3–36.0)	35.1 (32.8–36.2)	35.4 (32.3–36.3)	0.994
Parity				0.178
Nulliparous, *n* (%)	81 (60.9)	25 (53.2)	56 (65.1)	
Multiparous, *n* (%)	52 (39.1)	22 (46.8)	30 (34.9)	
Racial origin				0.968
White, *n* (%)	83 (62.4)	29 (61.7)	54 (62.8)	
Black, *n* (%)	38 (28.6)	14 (29.8)	24 (27.9)	
Other, *n* (%)	12 (9.0)	4 (8.5)	8 (9.3)	
Smoking, *n* (%)	1 (0.8)	0 (0.0)	1 (1.2)	0.458
Aspirin, *n* (%)	41 (30.8)	25 (53.2)	16 (18.6)	<0.001
BP at presentation				
Systolic (mmHg)	144.0 (138.2–149.0)	144.0 (137.7–148.0)	144.0 (140.0–149.5)	0.741
Diastolic (mmHg)	92.0 (87.0–94.7)	91.5 (87.7–95.2)	91.0 (87.0–93.0)	0.550
Diagnosis at presentation				0.689
GH, *n* (%)	79 (59.4)	29 (61.7)	50 (58.1)	
PET, *n* (%)	54 (40.6)	18 (38.3)	36 (41.9)	
Medication commenced at presentation				0.212
Beta blocker, *n* (%)	84 (63.2)	33 (70.2)	51 (59.3)	
Vasodilator, *n* (%)	49 (36.8)	14 (29.8)	35 (40.7)	
Angiogenic markers at presentation				
PlGF (pg/mL)	124.0 (65.2–274.3)	134.7 (80.4–351.8)	102.8 (58.5–191.7)	0.015
sFlt‐1 (pg/mL)	3719.8 (2049.8–6703.9)	2524.0 (1775.1–5713.3)	4304.3 (2434.4–7847.3)	0.063

*Note*: Data presented for the whole cohort and for two groups of women according to their BP control after 1 week: Group 1—BP <135/85, Group 2—BP ≥135/85. Data presented as median (interquartile range) for continuous and *n*(%) for categorical variables, and *p*‐values denote the differences between the two groups of women.

Abbreviations: BP, blood pressure, GH, gestational hypertension, PE, pre‐eclampsia, PLGF, placental growth factor, sFlt‐1, fms‐like tyrosine kinase‐1.

### 3.2 Mixed models analysis

**FIGURE 1:**
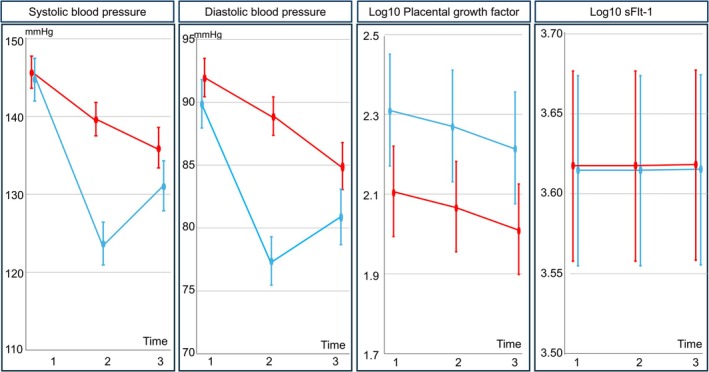
Linear mixed‐effects model with estimated marginal means and 95% confidence intervals for systolic blood pressure, diastolic blood pressure, Log_10_PLGF and Log_10_sFlt‐1 in women achieving therapeutic target within 1 week (blue line) or 2 weeks (red line) after commencement of antihypertensive treatment. Visit 1 corresponds to presentation, visit 2 after 1 week of treatment and visit 3 after 2 weeks of treatment.

The estimated marginal means and fixed and random effects of the multilevel models are shown in Table 2, Tables S1 and S2 and in Figure S1, Figure 1. For the multilevel models, only significant variables are presented.

**TABLE 2:** Multilevel linear mixed‐effect models for respective variables displayed as estimated marginal means with 95% confidence intervals.

	Visit 1	Visit 2	Visit 3
Systolic blood pressure			
Total cohort	145.3 (143.5–147.2)	133.5 *** (131.6–135.4)	134.2 *** (131.9–136.4)
Group 1	144.7 (141.9–147.5)	123.6 *** (120.8–126.3)	131.0 ***, +++ (127.9–134.2)
Group 2	145.7 (143.6–147.7)	139.6 *** (137.5–141.8)	135.9 ***, + (133.3–138.5)
Diastolic blood pressure			
Total cohort	90.9 (89.6–92.3)	84.3 *** (82.9–85.6)	83.1 *** (81.5–84.7)
Group 1	89.8 (87.9–91.8)	77.3 *** (75.4–79.2)	80.8 ***, + (78.6–83.0)
Group 2	91.9 (90.5–93.4)	88.9 ** (87.3–90.4)	84.9 ***, ++ (83.1–86.7)
Log_10_ PlGF			
Total cohort	2.123 (2.044–2.202)	2.084 * (2.005–2.163)	2.027 ***, ++ (1.946–2.109)
Group 1	2.309 (2.169–2.450)	2.270 (2.129–2.411)	2.213 ***, + (2.071–2.355)
Group 2	2.106 (1.992–2.220)	2.066 (1.952–2.181)	2.010 * (1.894–2.126)
Log_10_ sFlt‐1			
Total cohort	3.614 (3.555–3.673)	3.614 (3.555–3.673)	3.614 (3.555–3.673)
Group 1	3.614 (3.555–3.673)	3.614 (3.555–3.673)	3.614 (3.555–3.673)
Group 2	3.614 (3.555–3.673)	3.614 (3.555–3.673)	3.614 (3.555–3.673)

*Note*: Data presented for the whole cohort and for two groups of women according to their BP control after 1 week: Group 1—BP < 135/85, Group 2—BP ≥135/85. Compared to visit 1: *p < 0.05, **p < 0.01, ***p < 0.001; Compared to visit 2: +*p* < 0.05, ++*p* < 0.01, +++*p* < 0.00.

Abbreviations: PLGF, placental growth factor, sFlt‐1, fms‐like tyrosine kinase‐1.

For all variables, a random intercept model provided a significantly better fit to the data than did the base model or a random intercept‐random slope model.

#### 3.2.1 Maternal demographics—medication and diagnosis at presentation

##### Total cohort (Table S1)

Increasing gestational age was associated with an increase in DBP and Log_10_sFlt‐1. Increasing maternal weight was associated with a reduction in Log_10_sFlt‐1. The diagnosis of GH at presentation (as opposed to PE) was associated with lower SBP and Log_10_sFlt‐1. Nulliparity (as opposed to multiparity) was associated with higher DBP. Change over time (the 3 visits) was significant for SBP, DBP, and Log_10_PLGF, but not for Log_10_sFlt‐1. Maternal age and height, race, smoking status, aspirin or hypertensive agent (beta‐blocker or vasodilator) did not independently contribute to any of the models.

##### Group 1 versus group 2 (Table S2)

Increasing gestational age was associated with higher DBP and a lower Log_10_sFlt‐1. Increasing maternal height was associated with higher Log_10_PLGF. Increasing maternal weight was associated with a significant decrease in Log_10_sFlt‐1. Black and other racial groups were associated with increased Log_10_PLGF. The diagnosis of GH at presentation was associated with lower SBP. The time between visits was significant for SBP, DBP, and Log_10_PLGF. Maternal age, smoking status, aspirin, or type of hypertensive agent did not independently contribute to the model.

#### 3.2.2 Changes with time after controlling for maternal characteristics and outcome

##### Total cohort (Table 2, Figure S1)

In the total cohort, both SBP and DBP dropped after 1 week on antihypertensive medication with no significant change between visits 2 and 3. Log_10_PLGF demonstrated a continuous fall over the 2 weeks of treatment. On the contrary, Log_10_sFlt‐1 did not change with time after 1 and 2 weeks on medication.

##### Group 1 versus group 2 (Table 2, Figure 1)

In group 1, after 1 week of antihypertensive therapy there was a greater drop in SBP and DBP compared to group 2. After 2 weeks of treatment there was no significant difference in SBP and DBP between the two groups, as they had both achieved the therapeutic target. There was a continuous decrease in Log_10_PLGF in both groups over 2 weeks on treatment, with Group 2 having lower values than group 1. Log_10_sFlt‐1 did not change with time, with no difference between groups over the treatment period.

## 4 DISCUSSION

The results of our study demonstrate that in pregnant women with new onset hypertension, when assessing the whole cohort, tight BP control over 2 weeks leads to a reduction in SBP and DBP. Over the treatment period Log_10_PLGF declines but Log_10_sFlt‐1 shows no change with time. Women who achieved tight control after 1 week, compared to after 2 weeks, had persistently higher Log_10_PLGF but similar Log_10_sFlt‐1. There was a similar decline over time between groups for Log_10_PLGF but no change over time for Log_10_sFlt‐1.

In the literature, in vitro studies have yielded conflicting results regarding the effect of antihypertensive agents on angiogenic factors in cultured pregnancy cells. Studies in human umbilical vein endothelial cell culture have demonstrated inhibition of VEGF when treated with propranolol^19^ and upregulation with nifedipine.^21^ Methyldopa administered to human trophoblast cells reduced PLGF levels by 24%.^20^ On the contrary, Gangooly et al. who cultured placental villous explants and showed higher levels of sFlt‐1 in the culture from PE compared to non‐PE patients, did not find any effect on sFlt‐1 concentrations from labetalol, hydralazine, and methyldopa.^22^

In nonpregnant populations, one study of 17 chronic hypertensive women treated with enalapril for 2 months has demonstrated a favorable change in angiogenic profile with a reduction of VEGF and sFlt‐1. However, VEGF likely plays a key role in hypertensive disease outside of pregnancy and in a different context compared to that of PLGF in pregnancy. Treatment with VEGF inhibitors as part of cancer therapy has been observed to cause hypertension. A recent study looking at VEGF inhibitors to treat renal cell carcinoma reported that 66% of patients developed hypertension after treatment.^29^, ^30^ In pregnant populations, there is scant evidence on the effect of antihypertensive treatment on women's angiogenic profile. A small study demonstrated a reduction in sFlt‐1 in women with PE treated with methyldopa, but not in those with GH; this reduction was more pronounced in women with PE < 34 weeks (*n* = 28) compared to those with PE ≥34 weeks (*n* = 23).^16^ Similarly, in a study of 19 patients with PE, a decline in sFlt‐1 48‐h after treatment with methyldopa was reported, showing a dose‐dependent relationship.^17^

PLGF increases with gestation with a peak at 29–32 weeks and then falls until 37–41 weeks, with lower levels in those subsequently affected by PE and dramatically lower in those with established disease.^3^ In normotensive pregnancies sFlt‐1 remains stable until 33–36 weeks then starts to rise until delivery, whilst in those who were subsequently affected by PE this rise starts earlier at 21–24 weeks and reaches a higher concentration at 37–41 weeks. Again, patients with established disease have dramatically raised sFlt‐1 levels. In our study, PLGF levels declined with time, both in the total cohort and when comparing women who achieved target BP within 1 week or later. This is in keeping with the literature describing a reduction in PLGF after 29–32 weeks, and the fact that the median gestation of recruitment was 35 weeks gestation. However, although the rate of decline was the same in both groups, we do not have a control group of untreated hypertensive women to establish if the observed reduction with advancing gestational age is altered because of antihypertensive treatment. An interesting finding of our study was the fact that women who achieved target BP within 1 week had higher PLGF levels compared to those with more resistant hypertension and who needed 2 weeks to achieve target BP. This may indicate milder disease or a better cardiovascular system, more amenable to antihypertensive treatment. In any case, it is an observation that should lead to more research as to whether PLGF levels at presentation can triage hypertensive women and individualize the intensity of monitoring.

Contrary to PLGF, sFlt‐1 levels remained static during the study period, as opposed to the expected rise with increasing gestation. This suggests that tight BP control is associated with a suppression of the sFlt‐1 rise and supports the findings of Carr et al., who demonstrated an attenuated increase in sFlt‐1 in women at risk of PE treated with atenolol.^18^ An explanation of this finding could stem from the fact that tissue hypoxia is known to lead to upregulation of the Flt‐1 gene, leading to a predominant production of sFlt‐1.^31^ In hypertensive pregnant women, controlling BP reduces peripheral resistance and increases cardiac output,^32^, ^33^ which may improve tissue oxygenation and reduce sFlt‐1. Optimizing BP may also improve placental endothelial function by reducing shear stress and endothelial inflammation and injury.^18^ In addition, antihypertensives may influence the angiogenic profile independently of BP reduction. Mouse models have demonstrated that deactivation of a2‐adrenoceptors increased production of sFlt‐1, suggesting that their activation by alpha‐methyldopa may reduce sFlt‐1 levels.^34^

PLGF and sFlt‐1 have been shown to alter with gestation in both normotensive pregnancies and those affected by PE^3^ and to be affected by maternal demographics such as maternal weight, ethnicity, and cigarette smoking.^28^ Furthermore, as explained above, antihypertensive agents may have different effects on their concentrations. This is the reason why we controlled in our regression models for maternal demographics and type of antihypertensive medication, and we believe that this should be standard practice for anyone interpreting their changes in pregnancy.

Our study shows that tight BP control influences the angiogenic profile. This may reflect disease modification and therefore may, in the future, provide an alternative marker to measure treatment response. Lower PLGF was seen in women in whom it took longer to achieve tight BP control. PLGF therefore may be helpful in terms of determining intensity of follow up with women with lower PLGF being seen more frequently to ensure good BP control. However, the altered angiogenic profile must also be considered when interpreting sFlt‐1 and PLGF values in women already started on treatment as it may reduce their performance as predictive tools. The current literature uses a variety of cut offs of PLGF, sFlt‐1, and their ratio to predict progression to PE. The influence of antihypertensive and BP control on angiogenic factors should be factored in when calculating cut off thresholds to ensure validity.

This was the first study to assess longitudinal changes in both PLGF and sFlt‐1 in pregnant women commencing antihypertensive treatment aiming at tight BP control. Our study also had larger numbers than previous cross‐sectional studies and assessed both PLGF and sFlt‐1 levels rather than assessing sFlt‐1 alone.^16^, ^17^ Our study population had confirmed hypertensive disease rather than an at‐risk population, making the results clinically relevant and their demographics were heterogeneous, making these findings relatable to other populations.

A limitation of the study was the lack of a control group of women with untreated hypertension to compare their angiogenic profile. However, as optimal BP control is one of the cornerstones of current management of pregnancies with hypertensive disease, such an aim would not have been granted ethics approval.

Further work is required to assess whether sFlt‐1 and PLGF levels should be adjusted according to medication type and dosage and to see if there is a dose–response between antihypertensive treatment and effect on angiogenic parameter levels.

## 5 CONCLUSION

We have demonstrated that tight BP control alters the angiogenic profile of women with new‐onset hypertension in pregnancy. There is a steady decline in PLGF, as expected with advancing gestation. On the contrary, there is a clear attenuation of the expected rise in sFlt‐1. Women who took 2 weeks or more to achieve tight BP control had lower levels of PLGF but similar levels of sFlt‐1 at presentation. Changes over time in PLGF and sFlt‐1 are similar between women who achieved tight control over 1 week and those who needed two or more weeks.

## AUTHOR CONTRIBUTIONS

**Edward Tyrell:** Data curation, formal analysis, investigation, methodology, project administration, visualization, writing—original draft, writing—review and editing. **Katherine G. Y. Lau:** Data curation, investigation, methodology, project administration, writing—original draft. **Martyna Bednorz:** Data curation, investigation, methodology, writing—original draft. **Kypros H. Nicolaides:** Conceptualization, funding acquisition, investigation, methodology, resources, supervision, writing—review and editing. **Nikos A. Kametas:** Conceptualization, data curation, formal analysis, investigation, methodology, project administration, resources, supervision, validation, visualization, writing—original draft, writing—review and editing.

## FUNDING INFORMATION

The study was supported by a grant from the Fetal Medicine Foundation (Charity No: 1037116).

## CONFLICT OF INTEREST STATEMENT

The authors report no conflict of interest.

## ETHICS STATEMENT

This study received a favorable opinion from the Office of Research Ethics Committee Northern Ireland on January 22, 2018 (REC reference 18/NI/0013) and the National Research Ethics Service (NRES) Committee of East Midlands—Northampton on May 13, 2014 (REC Ref 14/EM/0141).

## Supporting information


**Figure S1.** Linear mixed‐effects model with estimated marginal means and 95% confidence intervals for systolic blood pressure, diastolic blood pressure, Log_10_PLGF, and Log_10_sFlt‐1 for the total cohort at presentation (visit 1), 1 week (visit 2), and 2 weeks (visit 3) after the commencement of antihypertensive treatment.

**Table S1.** Multilevel linear mixed‐effects models for respective variables for the total cohort: fixed and random effects. Only significant variables remaining in any of the final models are presented.

**Table S2.** Multilevel linear mixed‐effects models for respective variables. Fixed and random effects for two groups of women according to their BP control after 1 week: Group 1—BP <135/85, Group 2—BP ≥135/85. Only significant variables remaining in any of the final models are presented.

## Data Availability

The data that support the findings of this study are available on request from the corresponding author. The data are not publicly available due to privacy or ethical restrictions.

## References

[1] Duley L . The global impact of pre‐eclampsia and eclampsia. Semin Perinatol. 2009;33:130‐137. 19464502 10.1053/j.semperi.2009.02.010

[2] Herraiz I , Llurba E , Verlohren S , Galindo A . Update on the diagnosis and prognosis of preeclampsia with the aid of the sFlt‐1/ PlGF ratio in singleton pregnancies. Fetal Diagn Ther. 2018;43:81‐89. 28719896 10.1159/000477903

[3] Levine RJ , Maynard SE , Qian C , et al. Circulating angiogenic factors and the risk of preeclampsia. N Engl J Med. 2004;350:672‐683. 14764923 10.1056/NEJMoa031884

[4] Chaemsaithong P , Gil MM , Chaiyasit N , et al. Accuracy of placental growth factor alone or in combination with soluble fms‐like tyrosine kinase‐1 or maternal factors in detecting preeclampsia in asymptomatic women in the second and third trimesters: a systematic review and meta‐analysis. Am J Obstet Gynecol. 2023;229:222‐247. 36990308 10.1016/j.ajog.2023.03.032

[5] Welch PC , Amankwah KS , Miller P , Mcasey ME , Torry DS . Correlations of placental perfusion and PlGF protein expression in early human pregnancy. Am J Obstet Gynecol. 2006;194:1625‐1629. 16635470 10.1016/j.ajog.2006.01.012

[6] Bisson C , Dautel S , Patel E , Suresh S , Dauer P , Rana S . Preeclampsia pathophysiology and adverse outcomes during pregnancy and postpartum. Front Med (Lausanne). 2023;10:1144170. 37007771 10.3389/fmed.2023.1144170 PMC10060641

[7] Maynard SE , Min JY , Merchan J , et al. Excess placental soluble fms‐like tyrosine kinase 1 (sFlt1) may contribute to endothelial dysfunction, hypertension, and proteinuria in preeclampsia. J Clin Invest. 2003;111:649‐658. 12618519 10.1172/JCI17189 PMC151901

[8] Redman CWG , Staff AC , Roberts JM . Syncytiotrophoblast stress in preeclampsia: the convergence point for multiple pathways. Am J Obstet Gynecol. 2022;226:S907‐S927. 33546842 10.1016/j.ajog.2020.09.047

[9] Mckeeman GC , Ardill JE , Caldwell CM , Hunter AJ , Mcclure N . Soluble vascular endothelial growth factor receptor‐1 (sFlt‐1) is increased throughout gestation in patients who have preeclampsia develop. Am J Obstet Gynecol. 2004;191:1240‐1246. 15507947 10.1016/j.ajog.2004.03.004

[10] Dröge LA , Perschel FH , Stütz N , et al. Prediction of preeclampsia‐related adverse outcomes with the sFlt‐1 (soluble fms‐like tyrosine kinase 1)/PlGF (placental growth factor)‐ratio in the clinical routine: a real‐world study. Hypertension. 2021;77:461‐471. 33280406 10.1161/HYPERTENSIONAHA.120.15146

[11] Barton JR , Woelkers DA , Newman RB , et al. Placental growth factor predicts time to delivery in women with signs or symptoms of early preterm preeclampsia: a prospective multicenter study. Am J Obstet Gynecol. 2020;222:259.e1‐e11. 10.1016/j.ajog.2019.09.003 31518550

[12] Bergmann A , Ahmad S , Cudmore M , et al. Reduction of circulating soluble Flt‐1 alleviates preeclampsia‐like symptoms in a mouse model. J Cell Mol Med. 2010;14:1857‐1867. 19538465 10.1111/j.1582-4934.2009.00820.x PMC3829045

[13] Magee LA , Von Dadelszen P , Rey E , et al. Less‐tight versus tight control of hypertension in pregnancy. N Engl J Med. 2015;372:407‐417. 25629739 10.1056/NEJMoa1404595

[14] Tita AT , Szychowski JM , Boggess K , et al. Treatment for mild chronic hypertension during pregnancy. N Engl J Med. 2022;386:1781‐1792. 35363951 10.1056/NEJMoa2201295 PMC9575330

[15] Belgore FM , Blann AD , Li‐Saw‐Hee FL , Beevers DG , Lip GY . Plasma levels of vascular endothelial growth factor and its soluble receptor (SFlt‐1) in essential hypertension. Am J Cardiol. 2001;87:a9. 10.1016/s0002-9149(00)01512-5 11249912

[16] Khalil A , Muttukrishna S , Harrington K , Jauniaux E . Effect of antihypertensive therapy with alpha methyldopa on levels of angiogenic factors in pregnancies with hypertensive disorders. PLoS One. 2008;3:e2766. 18648513 10.1371/journal.pone.0002766 PMC2447877

[17] Herwati TW . Analysis of methyldopa therapy on sflt‐1 antiangiogenic levels in patients with severe preeclampsia. Folia Medica Indonesiana. 2018;2355‐8393:54.

[18] Carr DB , Tran LT , Brateng DA , et al. Hemodynamically‐directed atenolol therapy is associated with a blunted rise in maternal sFLT–1 levels during pregnancy. Hypertens Pregnancy. 2009;28:42‐55. 19165669 10.1080/10641950802132803

[19] Lamy S , Lachambre MP , Lord‐Dufour S , Béliveau R . Propranolol suppresses angiogenesis in vitro: inhibition of proliferation, migration, and differentiation of endothelial cells. Vasc Pharmacol. 2010;53:200‐208. 10.1016/j.vph.2010.08.002 20732454

[20] Bogacz A , Mikołajczak P , Wolek M , et al. Combined effects of methyldopa and flavonoids on the expression of selected factors related to inflammatory processes and vascular diseases in human placenta cells‐an in vitro study. Molecules. 2021;26:1259. 33652665 10.3390/molecules26051259 PMC7956652

[21] Luizon MR , Caldeira‐Dias M , Deffune E , et al. Antihypertensive therapy in pre‐eclampsia: effects of plasma from nonresponsive patients on endothelial gene expression. Pharmacogenomics. 2016;17:1121‐1127. 27348131 10.2217/pgs-2016-0033

[22] Gangooly S , Muttukrishna S , Jauniaux E . In‐vitro study of the effect of anti‐hypertensive drugs on placental hormones and angiogenic proteins synthesis in pre‐eclampsia. PLoS One. 2014;9:e107644. 25251016 10.1371/journal.pone.0107644 PMC4175458

[23] Snijders RJ , Nicolaides KH . Fetal biometry at 14‐40 weeks' gestation. Ultrasound Obstet Gynecol. 1994;4:34‐48. 12797224 10.1046/j.1469-0705.1994.04010034.x

[24] Robinson HP , Fleming JE . A critical evaluation of sonar “crown‐rump length” measurements. Br J Obstet Gynaecol. 1975;82:702‐710. 1182090 10.1111/j.1471-0528.1975.tb00710.x

[25] Muntner P , Shimbo D , Carey RM , et al. Measurement of blood pressure in humans: a scientific Statement from the American Heart Association. Hypertension. 2019;73:e35‐e66. 30827125 10.1161/HYP.0000000000000087 PMC11409525

[26] Clark K , Snowball O , Nzelu D , Kay P , Kametas NA . Validation of the microlife WatchBP home blood pressure device in pregnancy for medium and large arm circumferences. Blood Press Monit. 2018;23:171‐174. 29596069 10.1097/MBP.0000000000000315

[27] Magee LA , Brown MA , Hall DR , et al. The 2021 International Society for the Study of hypertension in pregnancy classification, diagnosis & management recommendations for international practice. Pregnancy Hypertens. 2022;27:148‐169. 35066406 10.1016/j.preghy.2021.09.008

[28] Tsiakkas A , Duvdevani N , Wright A , Wright D , Nicolaides KH . Serum soluble fms‐like tyrosine kinase‐1 in the three trimesters of pregnancy: effects of maternal characteristics and medical history. Ultrasound Obstet Gynecol. 2015;45:584‐590. 25678265 10.1002/uog.14817

[29] Brinda BJ , Viganego F , Vo T , Dolan D , Fradley MG . Anti‐VEGF‐induced hypertension: a review of pathophysiology and treatment options. Curr Treat Options Cardiovasc Med. 2016;18:33. 26932588 10.1007/s11936-016-0452-z

[30] Hall PS , Harshman LC , Srinivas S , Witteles RM . The frequency and severity of cardiovascular toxicity from targeted therapy in advanced renal cell carcinoma patients. JACC Heart Fail. 2013;1:72‐78. 24621801 10.1016/j.jchf.2012.09.001

[31] Lecarpentier E , Tsatsaris V . Angiogenic balance (sFlt‐1/PlGF) and preeclampsia. Ann Endocrinol (Paris). 2016;77:97‐100. 27130072 10.1016/j.ando.2016.04.007

[32] Stott D , Bolten M , Paraschiv D , Papastefanou I , Chambers JB , Kametas NA . Longitudinal hemodynamics in acute phase of treatment with labetalol in hypertensive pregnant women to predict need for vasodilatory therapy. Ultrasound Obstet Gynecol. 2017;49:85‐94. 27762457 10.1002/uog.17335

[33] Stott D , Papastefanou I , Paraschiv D , Clark K , Kametas NA . Serial hemodynamic monitoring to guide treatment of maternal hypertension leads to reduction in severe hypertension. Ultrasound Obstet Gynecol. 2017;49:95‐103. 27800645 10.1002/uog.17341

[34] Muthig V , Gilsbach R , Haubold M , et al. Upregulation of soluble vascular endothelial growth factor receptor 1 contributes to angiogenesis defects in the placenta of alpha 2B‐adrenoceptor deficient mice. Circ Res. 2007;101:682‐691. 17673674 10.1161/CIRCRESAHA.107.151563

