# Supplementary figures and images for: The effect of antihypertensive treatment on longitudinal changes in PLGF and sFlt‐1 in women with new onset hypertension in pregnancy
Source: Acta Obstet Gynecol Scand. 2026 May 13;105(8):1515–22. doi: 10.1111/aogs.70221 (PMC13356464; doi:10.1111/aogs.70221)

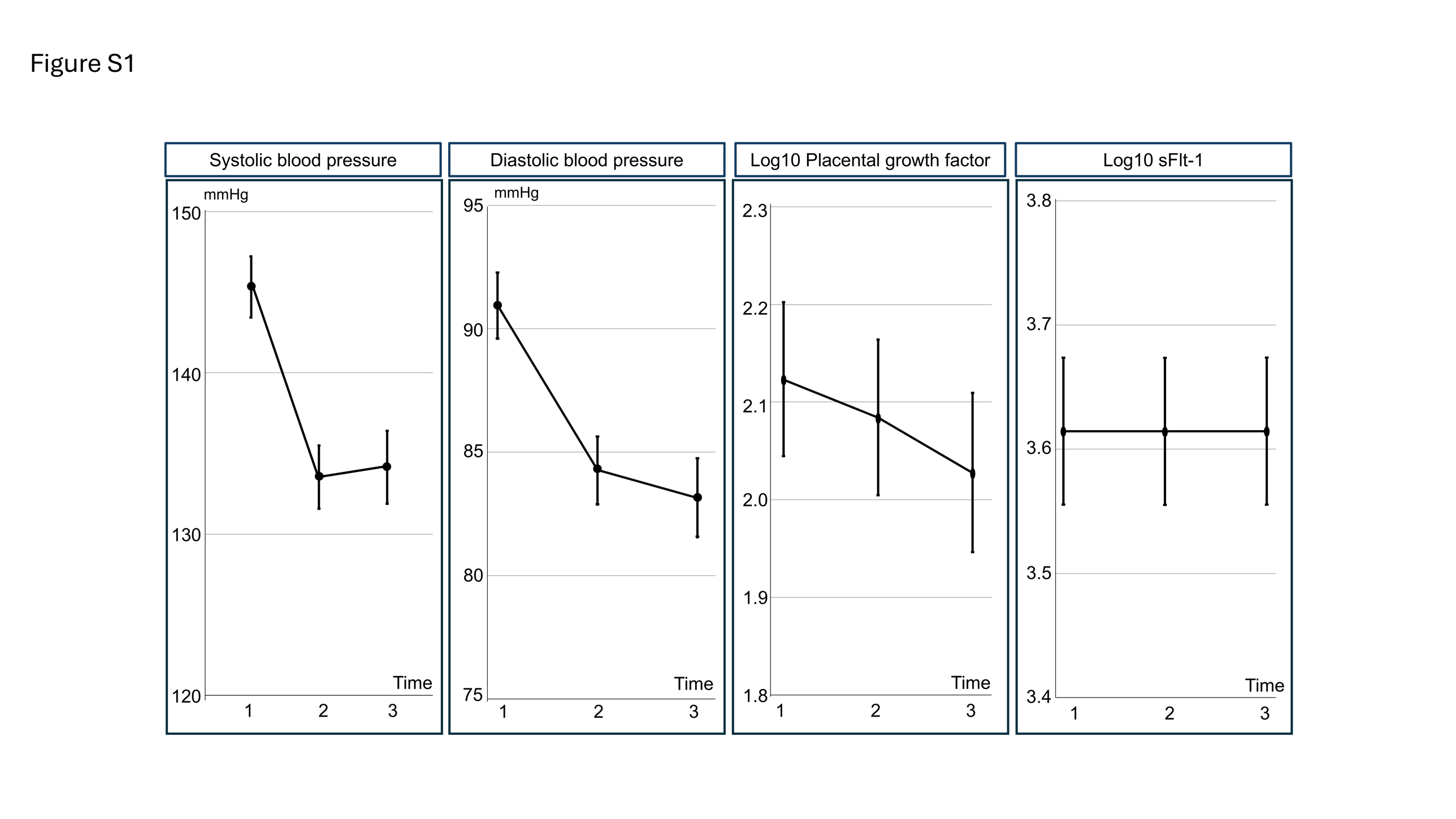

Supplement: Supplementary file 1 — Figure S1. Linear mixed‐effects model with estimated marginal means and 95% confidence intervals for systolic blood pressure, diastolic blood pressure, Log10PLGF, and Log10sFlt‐1 for the total cohort at presentation (visit 1), 1 week (visit 2), and 2 weeks (visit 3) after the commencement of antihypertensive treatment. [file AOGS-105-1515-s001.tif]
