# Supplementary material for: The effect of antihypertensive treatment on longitudinal changes in PLGF and sFlt‐1 in women with new onset hypertension in pregnancy
Source: Acta Obstet Gynecol Scand. 2026 May 13;105(8):1515–22. doi: 10.1111/aogs.70221 (PMC13356464; doi:10.1111/aogs.70221)
Supplement: Supplementary file 2 — Table S1. Multilevel linear mixed‐effects models for respective variables for the total cohort: fixed and random effects. Only significant variables remaining in any of the final models are presented. [file AOGS-105-1515-s002.docx]

| Table S1: Multilevel linear mixed-effects models for respective variables for the total cohort: fixed and random effects. Only significant variables remaining in any of the final models are presented. | | | | | | | | | |
| --- | --- | --- | --- | --- | --- | --- | --- | --- | --- |
| **Parameter** | **Systolic Blood Pressure** | | | **Diastolic Blood Pressure** | | | **Log_10_ Placental Growth Factor**  **(log_10_ PLGF)** | | |
| **Fixed effects parameters** | Estimate | Standard error | p-value | Estimate | Standard error | p-value | Estimate | Standard error | p-value |
| Intercept | 136.057 | 1.413 | < 0.001 | 63.007 | 5.978 | <0.001 | 2.027 | 0.041 | <0.001 |
| Gestational age |  |  |  | 0.548 | 0.164 | 0.001 |  |  |  |
| Maternal weight |  |  |  |  |  |  |  |  |  |
| Parity (reference multiparous) |  |  |  |  |  |  |  |  |  |
| Nulliparous |  |  |  | 2.263 | 1.001 | 0.025 |  |  |  |
| Diagnosis at presentation  (reference PET) |  |  |  |  |  |  |  |  |  |
| GH | -3.761 | 1.361 | 0.007 |  |  |  |  |  |  |
| Time (reference visit 3) |  |  |  |  |  |  |  |  |  |
| Visit 1 | 11.146 | 1.347 | < 0.001 | 7.802 | 0.950 | <0.001 | 0.095 | 0.019 | < 0.001 |
| Visit 2 | -0.636 | 1.366 | 0.642 | 1.131 | 0.936 | 0.228 | 0.056 | 0.020 | 0.005 |
| **Random effects parameters** |  |  |  |  |  |  |  |  |  |
| Variance of the constant per person | 22.756 | 8.441 | 0.007 | 14.702 | 4.179 | < 0.001 | 0.018 | 0.001 | < 0.001 |
| Variance of the residuals | 94.109 | 9.258 | < 0.001 | 43.346 | 4.174 | < 0.001 | 0.194 | 0.024 | < 0.001 |
|  |  |  |  |  |  |  |  |  |  |
| **Parameter** | **Log_10_ soluble fms-like tyrosine kinase-1 (Log_10_ sFlt-1)** | | |  |  |  |  |  |  |
| **Fixed effects parameters** | Estimate | Standard error | p-value |  |  |  |  |  |  |
| Intercept | 2.134 | 0.266 | <0.001 |  |  |  |  |  |  |
| Gestational age | 0.053 | 0.006 | <0.001 |  |  |  |  |  |  |
| Maternal weight | -0.003 | 0.002 | 0.039 |  |  |  |  |  |  |
| Parity (reference multiparous) |  |  |  |  |  |  |  |  |  |
| Nulliparous |  |  |  |  |  |  |  |  |  |
| Diagnosis at presentation  (reference PET) |  |  |  |  |  |  |  |  |  |
| GH | -0.198 | 0.059 | 0.001 |  |  |  |  |  |  |
| Time (reference visit 3) |  |  |  |  |  |  |  |  |  |
| Visit 1 |  |  |  |  |  |  |  |  |  |
| Visit 2 |  |  |  |  |  |  |  |  |  |
| **Random effects parameters** |  |  |  |  |  |  |  |  |  |
| Variance of the constant per person | 0.111 | 0.014 | <0.001 |  |  |  |  |  |  |
| Variance of the residuals | 0.011 | 0.001 | <0.001 |  |  |  |  |  |  |
