# Supplementary material for: The effect of antihypertensive treatment on longitudinal changes in PLGF and sFlt‐1 in women with new onset hypertension in pregnancy
Source: Acta Obstet Gynecol Scand. 2026 May 13;105(8):1515–22. doi: 10.1111/aogs.70221 (PMC13356464; doi:10.1111/aogs.70221)
Supplement: Supplementary file 3 — Table S2. Multilevel linear mixed‐effects models for respective variables. Fixed and random effects for two groups of women according to their BP control after 1 week: Group 1—BP <135/85, Group 2—BP ≥135/85. Only significant variables remaining in any of the final models are presented. [file AOGS-105-1515-s003.docx]

| Table S2: Multilevel linear mixed-effects models for respective variables. Fixed and random effects for two groups of women according to their BP control after one week: Group 1 – BP <135/85, Group 2 – BP ≥ 135/85. Only significant variables remaining in any of the final models are presented. | | | | | | | | | |
| --- | --- | --- | --- | --- | --- | --- | --- | --- | --- |
| **Parameter** | **Systolic Blood Pressure** | | | **Diastolic Blood Pressure** | | | **Log_10_ Placental Growth Factor**  **(log_10_ PLGF)** | | |
| **Fixed effects parameters** | Estimate | Standard error | p-value | Estimate | Standard error | p-value | Estimate | Standard error | p-value |
| Intercept | 132.861 | 1.765 | < 0.001 | 62.193 | 5.172 | <0.001 | 0.073 | 0.953 | 0.939 |
| Gestational age |  |  |  | 0.537 | 0.141 | <0.001 |  |  |  |
| Maternal height |  |  |  |  |  |  | 0.011 | 0.006 | 0.044 |
| Maternal weight |  |  |  |  |  |  |  |  |  |
| Race (reference White) |  |  |  |  |  |  |  |  |  |
| Black |  |  |  |  |  |  | 0.192 | 0.083 | 0.022 |
| Other |  |  |  |  |  |  | 0.244 | 0.134 | 0.072 |
| Diagnosis at presentation  (reference PET) |  |  |  |  |  |  |  |  |  |
| GH | -3.617 | 1.189 | 0.003 |  |  |  |  |  |  |
| Time (reference visit 3) |  |  |  |  |  |  |  |  |  |
| Visit 1 | 13.645 | 1.964 | < 0.001 | 9.021 | 1.373 | <0.001 | 0.096 | 0.019 | < 0.001 |
| Visit 2 | -7.467 | 1.964 | < 0.001 | -3.504 | 1.351 | 0.010 | 0.057 | 0.020 | 0.005 |
| Group (reference Group 1) |  |  |  |  |  |  |  |  |  |
| Group 2 | 4.872 | 2.061 | 0.019 | 4.106 | 1.447 | 0.005 | -0.244 | 0.134 | 0.072 |
| Interaction (group x visit) |  |  | <0.001 |  |  | <0.001 |  |  |  |
| **Random effects parameters** |  |  |  |  |  |  |  |  |  |
| Variance of the constant per person | 14.543 | 6.605 | 0.028 | 9.309 | 3.248 | < 0.001 | 0.174 | 0.023 | < 0.001 |
| Variance of the residuals | 79.114 | 7.822 | < 0.001 | 37.156 | 3.248 | 0.004 | 0.018 | 0.002 | < 0.001 |
|  |  |  |  |  |  |  |  |  |  |
|  |  |  |  |  |  |  |  |  |  |

| Table S2 continued: Multilevel linear mixed-effects models for respective variables. Fixed and random effects for two groups of women according to their BP control after one week: Group 1 – BP <135/85, Group 2 – BP ≥ 135/85. Only significant variables remaining in any of the final models are presented. | | | | |
| --- | --- | --- | --- | --- |
| **Parameter** | **Log_10_ soluble fms-like tyrosine kinase-1 (Log_10_ sFlt-1)** | | |  |
| **Fixed effects parameters** | Estimate | Standard error | p-value |  |
| Intercept | 2.135 | 0.266 | <0.001 |  |
| Gestational age | -0.198 | 0.060 | 0.001 |  |
| Maternal height |  |  |  |  |
| Maternal weight | -0.003 | 0.002 | 0.039 |  |
| Ethnicity (reference White) |  |  |  |  |
| Black |  |  |  |  |
| Other |  |  |  |  |
| Diagnosis at presentation  (reference PET) |  |  |  |  |
| GH |  |  |  |  |
| Medication (reference Beta Blockers) |  |  |  |  |
| Vasodilators |  |  |  |  |
| Time (reference visit 3) |  |  |  |  |
| Visit 1 |  |  |  |  |
| Visit 2 |  |  |  |  |
| Group (reference Group 1) |  |  |  |  |
| Group 2 |  |  |  |  |
| Interaction |  |  |  |  |
| **Random effects parameters** |  |  |  |  |
| Variance of the constant per person | 0.111 | 0.014 | <0.001 |  |
| Variance of the residuals | 0.011 | 0.001 | <0.001 |  |
